# Precision diagnosis model for treatment-resistant depression integrating serum metabolomics and clinical risk factors

**DOI:** 10.3389/fpsyt.2026.1826019

**Published:** 2026-05-21

**Authors:** Weiquan Huang, Xiujuan Hong, Chenjunyi Huang, Mengye Gao, Lanqing Li, Qinqin Lou, Junli Gao, Junshun Gao

**Affiliations:** 1 Laboratory Department of Huzhou Third Municipal Hospital, the Affiliated Hospital of Huzhou University, Huzhou, China; 2 Zhejiang Chinese Medical University, Hangzhou, China; 3 Cosmos Wisdom Mass Spectrometry Center of Zhejiang University Medical School, Hangzhou, China

**Keywords:** diagnostic model, machine learning, metabolomics, risk factors, treatment-resistant depression

## Abstract

**Objective:**

This study aimed to construct a high-efficiency dual-modal diagnostic model for treatment-resistant depression (TRD) by integrating serum metabolomics and clinical risk factors, and explore its metabolic pathological mechanisms.

**Methods:**

A total of 93 major depressive disorder (MDD) patients (53 TRD, 40 non-TRD) were enrolled for a single-center retrospective study. Serum untargeted metabolomics and clinical baseline data were collected, with differential metabolites and clinical risk factors screened by statistical analysis and multi-step machine learning to identify core features. Five machine learning algorithms were compared to build unimodal and random forest-based dual-modal diagnostic models, and KEGG pathway enrichment analysis was performed.

**Results:**

3 core clinical risk factors (medical history, HDL, FBG) and 8 core metabolic biomarkers were identified. The dual-modal model achieved AUC 0.996 (training) and 0.911 (validation), outperforming unimodal models. Differential metabolites were mainly enriched in lipid (44.8%) and amino acid (23.9%) metabolism. Fibrinopeptide A516, 12-HETE and the three clinical factors were core driving features.

**Conclusion:**

The dual-modal model has high diagnostic efficiency for TRD. TRD is associated with endocannabinoid system hypofunction and metabolic imbalance, which provides an objective diagnostic tool and new insights for TRD mechanism research and therapy development.

## 1 Introduction

Major depressive disorder (MDD) is a prevalent and debilitating psychiatric disorder characterized by persistent low mood, anhedonia, and impaired social and occupational function, imposing a heavy burden on global public health and healthcare systems (1). Despite the availability of a variety of antidepressant medications and therapeutic strategies, approximately 30%-50% of MDD patients fail to achieve satisfactory clinical responses after adequate courses of first-line antidepressant treatment and are diagnosed with treatment-resistant depression (TRD) (2). The identification and early intervention of TRD are critical for improving treatment outcomes and reducing the chronicity and recurrence of depression; however, the clinical diagnosis of TRD currently relies solely on subjective symptom evaluation and treatment response assessment, lacking objective and specific biological indicators to support differential diagnosis from non-TRD MDD (3). This subjectivity not only leads to delayed diagnosis and inappropriate treatment allocation for TRD patients but also hinders the in-depth exploration of the underlying pathological mechanisms of treatment resistance.

In recent years, metabolomics has emerged as a powerful tool for investigating the molecular mechanisms of psychiatric disorders and screening potential biomarkers, by characterizing the comprehensive metabolic profiles of biological samples and reflecting the dynamic changes of the body’s metabolic network in response to pathological states. Abnormalities in lipid metabolism, amino acid metabolism, and inflammatory metabolic pathways have been repeatedly reported in MDD patients, and these metabolic perturbations are thought to be closely associated with the pathogenesis of depression and the variability of treatment responses (4). For TRD, preliminary metabolomic studies have revealed distinct metabolic characteristics compared with non-TRD MDD, suggesting that metabolic biomarkers may serve as objective indicators for the differential diagnosis of TRD (5, 6).

Clinical risk factors, including chronic disease history, metabolic indicators, and biochemical parameters, have been shown to be associated with the occurrence and development of TRD (7). The combination of metabolomic biomarkers with clinical risk factors can complement the advantages of both, capture more comprehensive pathological information of TRD, and thus improve the accuracy of diagnostic models. Machine learning algorithms, as an emerging data analysis method, have been widely used in the construction of diagnostic models for complex diseases, and can effectively screen core predictive features and optimize model performance from high-dimensional data. Among them, the random forest algorithm has the advantages of strong anti-overfitting ability, high generalization performance, and insensitivity to high-dimensional data, making it suitable for the construction of multi-modal integrated diagnostic models for TRD (8).

In this study, we enrolled 93 MDD patients (53 TRD cases and 40 non-TRD MDD cases) and integrated untargeted metabolomic profiling of serum samples with clinical baseline data analysis. On this basis, we constructed a dual-modal joint diagnostic model integrating metabolic biomarkers and clinical risk factors based on the machine learning algorithm. We demonstrated that the dual-modal diagnostic model combining metabolic biomarkers and clinical risk factors would exhibit higher diagnostic efficiency than single-modal models, and the identified differential metabolites would reveal key metabolic pathways involved in TRD, providing new insights for the objective diagnosis and pathological mechanism research of TRD.

## 2 Materials and methods

### 2.1 Study design

**Figure 1:**
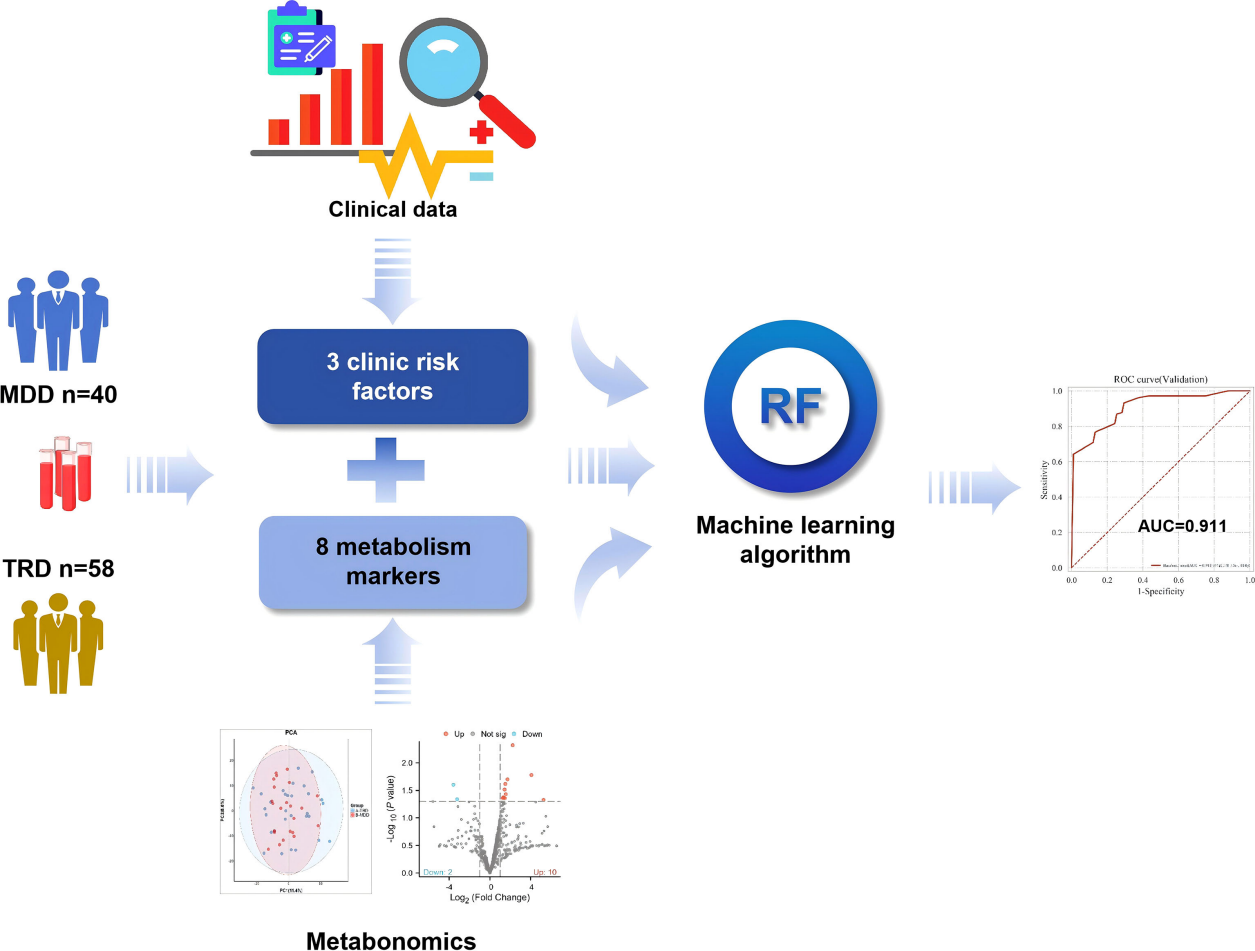
Schematic overview of the study design and analytical workflow.

A single-center retrospective study included 93 eligible MDD patients (53 TRD, 40 non-TRD MDD) after quality control (**Figure 1**). Integrated serum untargeted metabolomics and clinical data analysis, via machine learning for feature selection and model construction, we built a dual-modal TRD diagnostic model, and explored potential metabolic mechanisms by KEGG pathway analysis. This study has been approved by Huzhou Third People’s Hospital Medical Ethics Committee (No. 2023-376), and all data containing patient identity information have been de-labeled.

### 2.2 Patients

MDD Diagnosis: All patients met the diagnostic criteria for MDD as defined by the Diagnostic and Statistical Manual of Mental Disorders, Fifth Edition (DSM-5). Diagnosis was confirmed by two senior psychiatrists through structured clinical interviews.

TRD Definition: TRD was defined as failure to achieve a ≥50% reduction in depressive symptoms (measured by the 17-item Hamilton Depression Rating Scale [HAMD-17]) after at least two adequate courses (≥6 weeks each) of different antidepressant medications at therapeutic doses.

MDD Group: Patients who achieved a ≥50% reduction in HAMD-17 scores after one adequate course of antidepressant treatment and did not meet the TRD criteria.

Exclusion Criteria: Comorbid with other psychiatric disorders; Severe physical diseases (malignant tumors, severe liver/kidney dysfunction, acute cardiovascular events); History of substance abuse or dependence (alcohol, drugs) within the past 6 months; Pregnant or lactating women; Recent use of medications affecting metabolic profiles within 4 weeks; Incomplete clinical data or metabolomics samples that failed quality control.

### 2.3 Clinical data collection

A standardized case report form was used to collect clinical data from all participants, including demographic characteristics [age, gender, body mass index (BMI)], clinical features (disease duration, HAMD-17 score, antidepressant treatment history), and laboratory indicators.

### 2.4 Sample collection and processing

Fasting venous blood (5 mL) was collected from each participant between 8:00 and 10:00 a.m. after an overnight fast (≥12 hours).

Sample Processing: Blood samples were centrifuged at 3, 000 × g for 15 minutes at 4°C to separate serum. The supernatant serum was aliquoted into 1.5 mL RNase/DNase-free cryopreservation tubes and immediately stored at -80°C until metabolomics analysis. To avoid repeated freeze-thaw cycles, each aliquot was used only once for detection.

### 2.5 Non-targeted metabolomics analysis

Untargeted metabolomic analysis was performed on the CalOmics metabolomics platform of Calibra Scientific, Inc. using ACQUITY 2D UPLC (Waters, USA) coupled with Q Exactive hybrid Quadrupole-Orbitrap mass spectrometer (Thermo Fisher Scientific, USA). LC-MS grade methanol, acetonitrile, formic acid and other reagents (Sigma-Aldrich, USA) were used for the experiment. Serum samples were mixed with methanol at a volume ratio of 1:4, oscillated for 3 min, and centrifuged at 4000 × g for 10 min at 20 °C to precipitate proteins. The supernatant was aliquoted, dried under nitrogen blowing, reconstituted with the corresponding solution, and detected by four UPLC-MS/MS methods (positive/negative ion modes with C18/HILIC column separation). The mass spectrometer was set at a resolution of 35000 and a scan range of 70–1000 m/z.

Raw metabolomics data were subjected to strict quality control first. Ion peak extraction and metabolite identification were completed using the platform’s proprietary in-house data processing system and self-built metabolite database (matching criteria: retention index, accurate mass variation < 5 ppm, MS/MS fragmentation spectra with cross-referencing score > 0.7). Metabolite quantification was based on the area under the chromatographic peak curve.Data normalization was performed as follows: log2 transformation of raw peak areas, median normalization, and imputation of missing values with the minimum detection value of the corresponding metabolite. Differential metabolites were screened by univariate tests (Welch’s t-test/Wilcoxon rank-sum test) with Benjamini-Hochberg false discovery rate (FDR) correction (q<0.05), combined with multivariate analysis (PCA/OPLS-DA) criteria of VIP > 1 and |log2(FC)| > 0.58. KEGG pathway enrichment analysis of differential metabolites was conducted via MetPA and Pathview toolkits with hypergeometric test for significance verification. All statistical analyses and data visualization were completed using R software (v3.4.1) with mixOmics, randomForest and other key packages.

### 2.6 Feature selection

Differential metabolite screening (univariate tests and OPLS-DA VIP > 1) was performed strictly within the training set of the primary 7:3 split; PCA/OPLS-DA score plots were generated from the full cohort for visualization only and were not used for feature selection. A total of 12 significantly differential metabolites were initially identified, and Boruta algorithm was used for iterative feature selection to obtain 10 candidate metabolic markers (green features). The random forest model was then applied to evaluate the importance of the 10 candidate markers, and eXtreme Gradient Boosting (XGBoost) was used for variable set scoring. The results showed that the model AUC reached a peak when 8 metabolic markers were included, and these 8 markers were finally locked as core metabolic features for subsequent modeling.

3 clinical risk factors with significant intergroup differences (TRD vs. MDD) were screened via univariate comparison of clinical indicators, and were directly included in the subsequent model construction.

To verify that the selected clinical variables carried independent predictive information beyond univariate associations, we performed LASSO-regularized multivariable logistic regression (L1 penalty, 5-fold cross-validation for lambda selection) on the combined metabolic and clinical dataset. Variables retaining non-zero coefficients after LASSO shrinkage were considered independent predictors.

### 2.7 Machine learning model construction and validation

The integrated dataset was split into a training set and a validation set by stratified random sampling to maintain the consistent ratio (7:3) of TRD/MDD patients in both subsets and avoid selection bias.

Two single-modal datasets (8 metabolic markers, 3 clinical risk factors) were constructed respectively, and 5 machine learning algorithms were applied for modeling and performance comparison for each modality. Five machine learning algorithms (Logistic Regression, SVM, LightGBM, XGBoost, Random Forest) were constructed and optimized by 5-fold cross-validation. The optimal algorithm for each single-modal model was screened based on the validation set area under the receiver operating characteristic curve (AUC), sensitivity, specificity and generalization ability.

Based on the optimal algorithm screened from single-modal modeling, a dual-modal joint detection model was constructed by integrating the 8 metabolic markers and 3 clinical risk factors. Hyperparameters of the dual-modal model were optimized via 5-fold cross-validation on the training set to minimize overfitting.

To further assess the robustness of the model and address potential overfitting concerns inherent to multi-step feature selection, we conducted additional secondary validation analyses: (i) nested 5×3-fold cross-validation with strict separation of feature selection and performance estimation; (ii) bootstrap resampling (n=100) to evaluate feature selection stability; (iii) LASSO-regularized multivariable logistic regression to verify the independence of clinical predictors after adjusting for metabolic covariates; and (iv) decision curve analysis (DCA) and calibration assessment to evaluate clinical utility.

### 2.8 Model performance evaluation

Model diagnostic performance was comprehensively evaluated using AUC, sensitivity, specificity and overall accuracy. ROC curves were plotted using the R pROC package, and the 95% confidence intervals (CI) of AUC values were calculated via the bootstrap method (1, 000 iterations). A two-sided *P* < 0.05 was considered statistically significant for all analyses.

SHAP (SHapley Additive exPlanations) analysis was performed using the Python shap package to interpret the final dual-modal model. SHAP summary plots and mean absolute SHAP value bar charts were generated to visualize the direction and magnitude of each feature’s impact on the model’s prediction results, and to identify the core driving features of the model.

### 2.9 Statistical analysis

Statistical analyses were performed using R (v4.3.1) and Python (v3.10). A two-sided *P* < 0.05 was considered statistically significant. The statistical analysis was all processed on SPSS 26.0. All the data were tested using the normal distribution test. The t-test tested the data in accordance with the normal distribution, and the data out accordance with the normal distribution were tested by the non-parametric test. Mann-Whitney U test was used to compare two groups, and the KruskalWallis test was used to compare three or more groups. A P-value less than 0.05 was considered statistically significant.

## 3 Results

### 3.1 Clinical baseline characteristics of the study population

A total of 93 patients with MDD were included in the final analysis (40 non-TRD cases and 53 TRD cases), with baseline clinical data compared between the two groups (**Table 1**). No statistically significant differences were observed in gender distribution, age, disease course, C-reactive protein (CRP), triglyceride (TG), total cholesterol, low-density lipoprotein cholesterol (LDL), systolic blood pressure (SBP), and diastolic blood pressure (DBP) between the TRD and non-TRD MDD groups (all *P* > 0.05). In contrast, three clinical indicators showed significant intergroup differences: medical history (Z=-3.81, *P* < 0.001), high-density lipoprotein cholesterol (HDL) (Z=-3.45, *P* < 0.001), and fasting blood glucose (FBG) (Z=-2.21, *P* = 0.027). These three differentially expressed clinical indicators were identified as core clinical risk factors and directly incorporated into subsequent model construction. LASSO-regularized multivariable regression independently retained all three clinical variables (medical history, HDL, FBG) with non-zero coefficients after adjusting for metabolic covariates, confirming their status as independent rather than confounded predictors (**Supplementary Table 2**).

**Table 1:** Clinical baseline analysis of 93 patients with MDD.

Variables	Total (n = 93)	MDD (n = 40)	TRD (n = 53)	Statistic	P
Gender, n(%)				χ²=1.75	0.186
Male	16 (17.20)	5 (12.50)	11 (20.75)		
Female	77 (82.80)	35 (87.50)	42 (79.25)		
Age, M (Q_1_, Q_3_)	37.00 (22.00, 52.00)	35.00 (18.00, 52.00)	37.00 (22.50, 51.75)	Z=-0.40	0.689
Medical history (years), M (Q_1_, Q_3_)	3.00 (1.12, 10.00)	2.00 (1.00, 4.50)	7.00 (2.00, 12.25)	Z=-3.81	<.001
Course of the disease (days), M (Q_1_, Q_3_)	16.50 (8.50, 30.00)	30.00 (20.25, 30.00)	15.00 (8.00, 30.25)	Z=-1.13	0.258
CRP, M (Q_1_, Q_3_)	1.06 (0.39, 2.36)	0.58 (0.35, 1.77)	1.66 (0.43, 2.64)	Z=-1.50	0.134
TG, M (Q_1_, Q_3_)	1.25 (0.87, 1.73)	1.26 (0.88, 1.63)	1.23 (0.88, 2.13)	Z=-0.58	0.564
Total cholesterol, M (Q_1_, Q_3_)	4.85 (4.21, 5.54)	5.16 (4.50, 5.57)	4.62 (4.11, 5.43)	Z=-1.39	0.166
HDL, M (Q_1_, Q_3_)	1.29 (1.08, 1.60)	1.47 (1.25, 1.67)	1.21 (0.98, 1.35)	Z=-3.45	<.001
LDL, M (Q_1_, Q_3_)	2.71 (2.16, 3.03)	2.72 (2.26, 3.08)	2.68 (2.10, 2.95)	Z=-0.25	0.799
SBP, M (Q_1_, Q_3_)	108.00 (103.00, 120.00)	134.00 (134.00, 134.00)	108.00 (102.75, 119.25)	Z=-1.24	0.215
DBP, M (Q_1_, Q_3_)	70.00 (67.00, 82.00)	68.00 (68.00, 68.00)	71.50 (66.75, 84.00)	Z=-0.50	0.620
FBG, M (Q_1_, Q_3_)	5.04 (4.69, 5.69)	5.20 (4.77, 6.33)	4.89 (4.59, 5.30)	Z=-2.21	0.027

### 3.2 Untargeted metabolomics reveals distinct metabolic profiles between TRD and MDD groups

**Figure 2:**
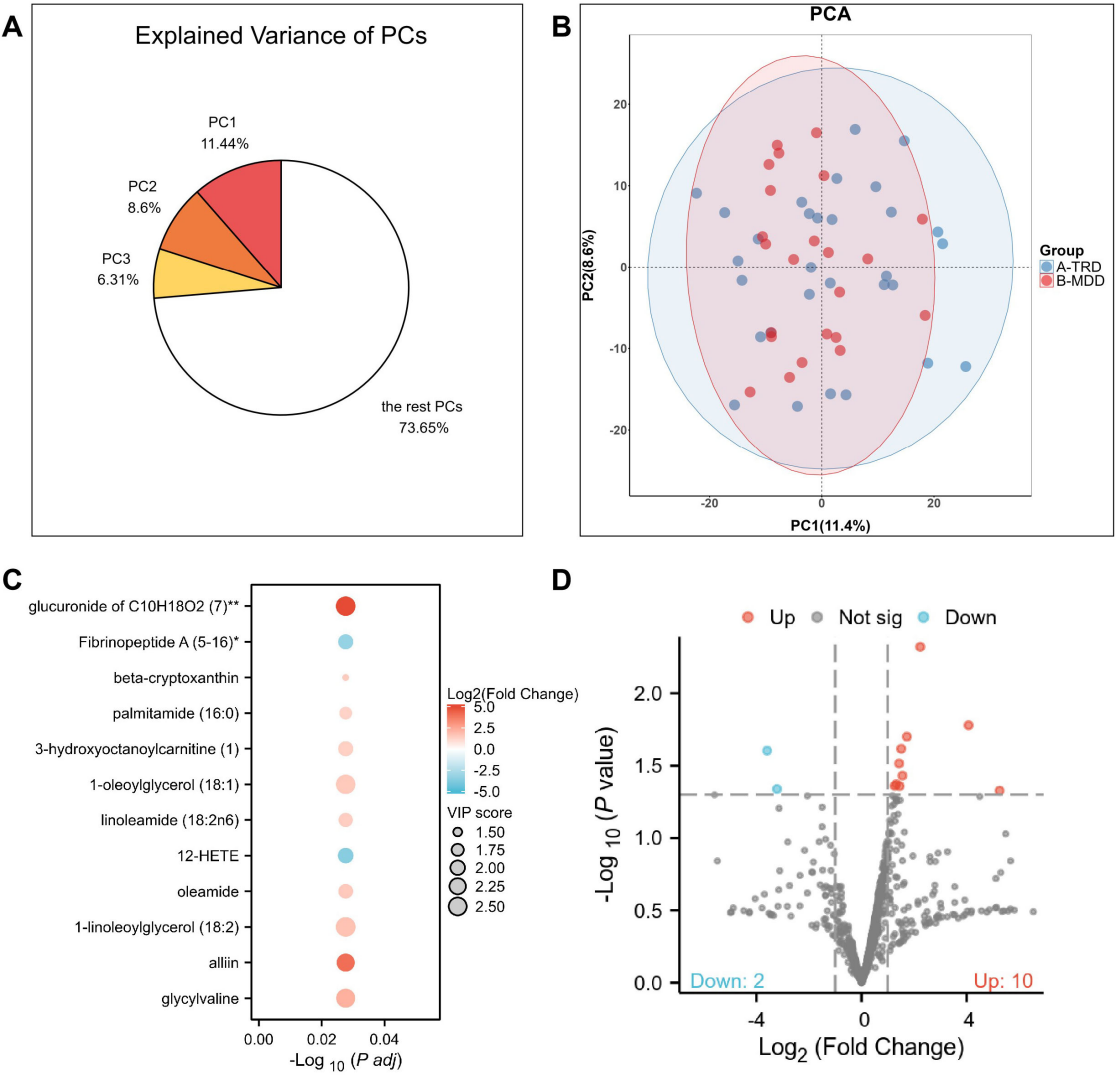
Metabolomics profiling and differential analysis between MDD and TRD groups. **(A)** Explained variance of the principal components (PCs) from principal component analysis (PCA). **(B)** PCA score plot showing the separation between MDD (red) and TRD (blue) groups. **(C)** VIP (variable importance in projection) plot from OPLS-DA, ranking the top 12 metabolites by their importance in group separation. **(D)** Volcano plot illustrating the distribution of metabolites based on log2(fold change) and -log10(*P* value). Red dots represent significantly upregulated metabolites (n=10), blue dots represent significantly downregulated metabolites (n=2), and gray dots represent non-significant metabolites.

Serum samples from all patients were subjected to untargeted metabolomic profiling using ACQUITY 2D UPLC-Q Exactive high-resolution mass spectrometry, with multivariate and univariate analyses performed to characterize metabolic differences (**Figure 2**). Principal component analysis (PCA) score plots exhibited a clear separation trend in the global metabolic profiles between the TRD and MDD groups, indicating intrinsic metabolic heterogeneity between the two cohorts. OPLS-DA model validation by 200 permutation tests yielded R²Y = 0.89, Q² = 0.42, and permutation p < 0.001, confirming that the observed group separation was statistically robust and not attributable to overfitting. Volcano plot analysis (with screening criteria of FDR q < 0.05, VIP > 1, and |log2(FC)| > 0.58) identified 12 significantly differential metabolites between the two groups. After Benjamini-Hochberg FDR correction, 11 metabolites remained significant at q < 0.05, including all 8 core metabolic biomarkers that entered the final model. Among these, 10 metabolites were upregulated and 2 were downregulated in the TRD group relative to the MDD group, which were defined as potential metabolic biomarkers for TRD and further subjected to rigorous feature screening.

### 3.3 Serum levels of candidate metabolic biomarkers in TRD and MDD groups

**Figure 3:**
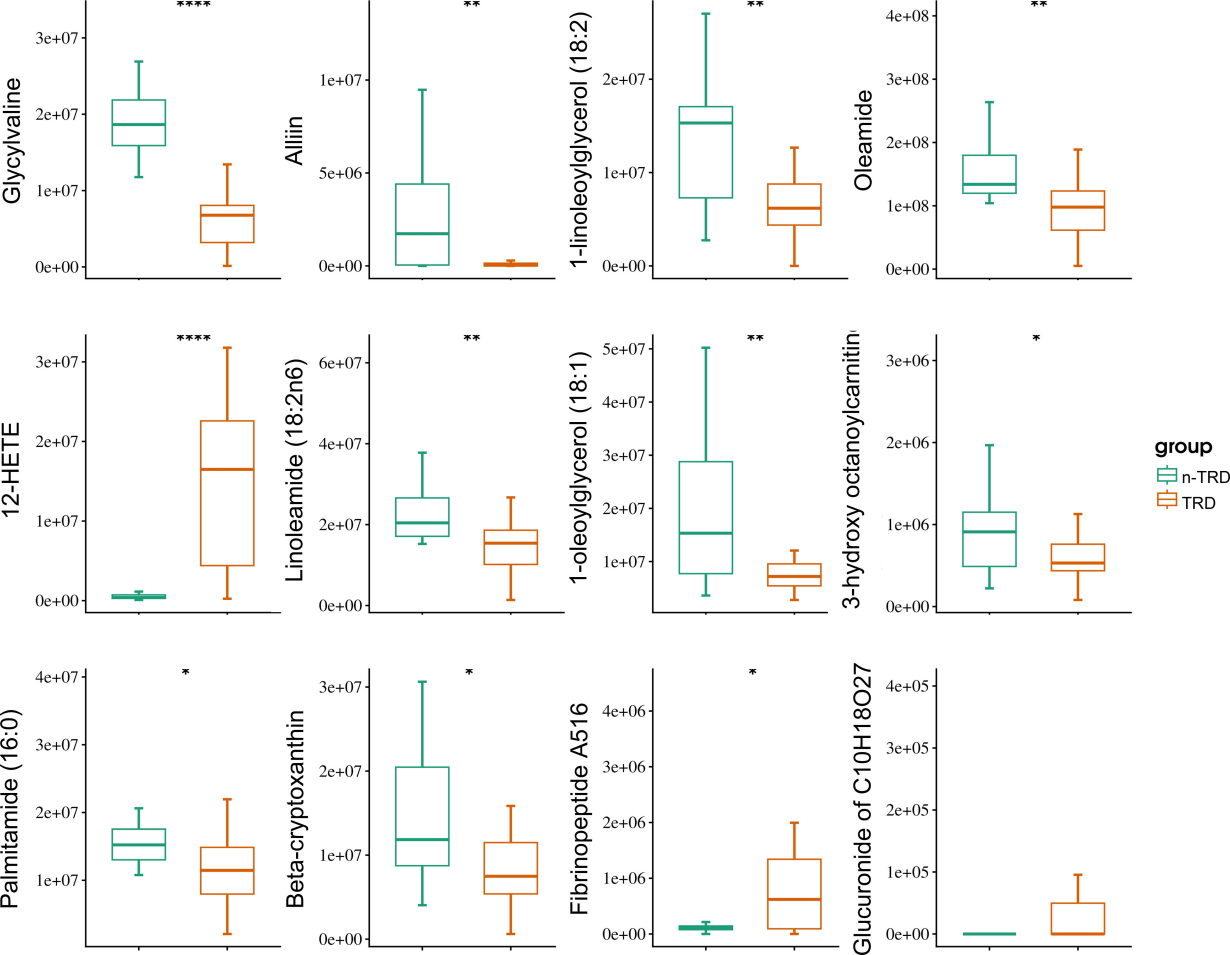
Serum level of candidate metabolic markers. Box plots showing the relative abundance of 12 candidate metabolic markers in n-TRD (green) and TRD (orange) groups. Significance levels are indicated as follows: *P < 0.05, **P < 0.01, ****P < 0.0001 (Student’s t-test or Mann-Whitney U test).

The 12 differentially screened metabolites were further validated for their expression levels in the TRD and MDD groups (**Figure 3**). Box plots of relative serum abundances confirmed that all 12 candidate metabolic biomarkers exhibited statistically significant differences between the two groups (all *P* < 0.05). Specifically, 12-HETE, Fibrinopeptide A516, and Glucuronide of C10H18O2 were significantly upregulated in the TRD group, while oleamide, linoleamide (18:2n6), palmitamide (16:0), 1-linoleoylglycerol (18:2), 1-oleoylglycerol (18:1), Alliin, Glycylvaline, 3-hydroxy octanoylcarnitine, and beta-cryptoxanthin were significantly downregulated. These results verified the robustness of the metabolomic differences and provided a quantitative basis for subsequent feature selection and model construction.

### 3.4 Machine learning-based feature selection identifies core metabolic biomarkers

**Figure 4:**
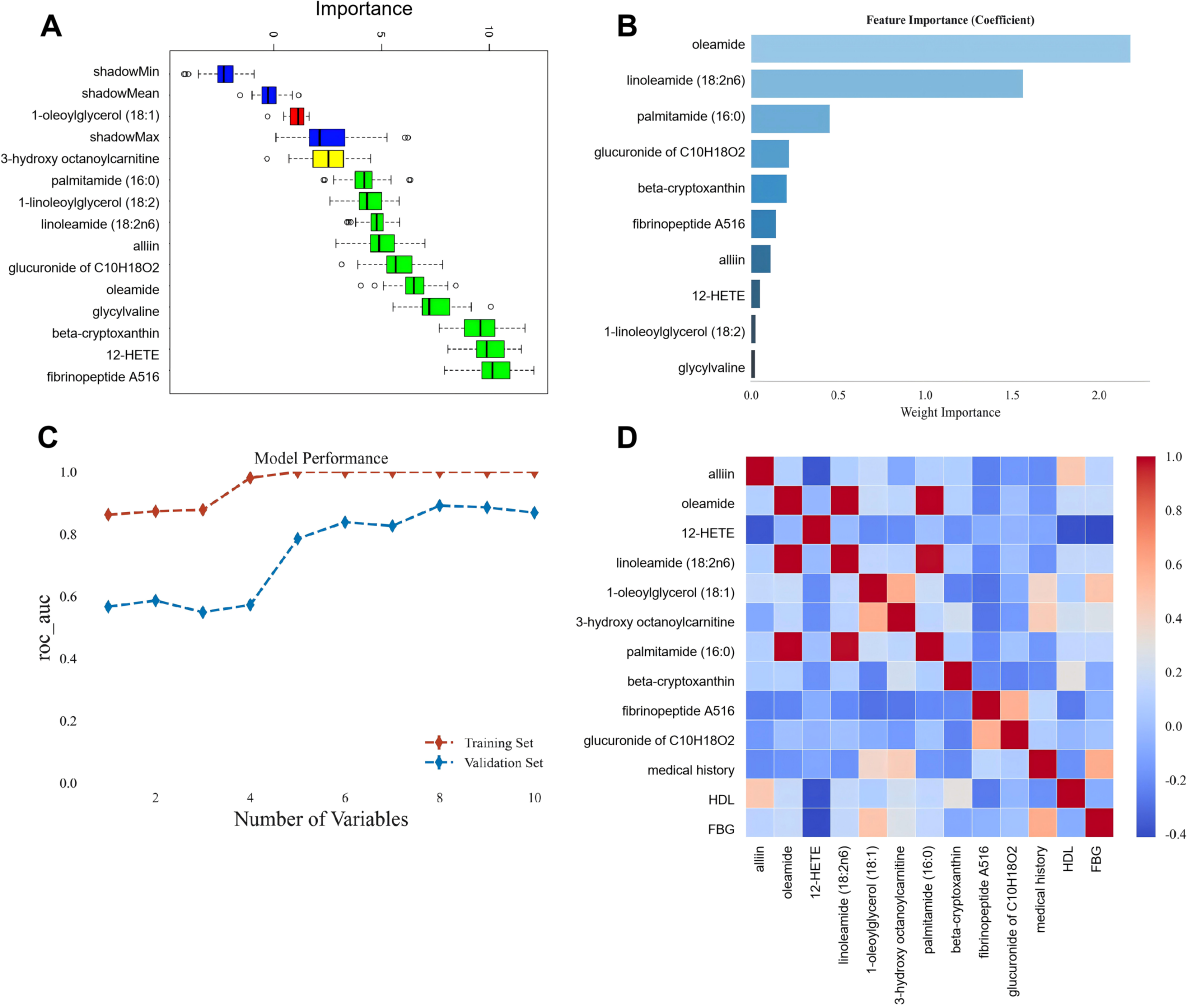
Multi-strategy screening of core metabolic biomarkers for TRD. **(A)** Variable importance ranking of 10 candidate metabolites filtered from 12 differential metabolites by Boruta algorithm; **(B)** XGBoost variable set scoring for the 10 candidate metabolites; **(C)** Model performance curve showing AUC changes with incremental addition of ranked metabolic variables (AUC peaked at 8 metabolites in both training and validation sets); **(D)** Correlation heatmap between the final 8 core metabolic biomarkers (oleamide, linoleamide (18:2n6), palmitamide (16:0), glucuronide of C10H18O2, beta-cryptoxanthin, fibrinopeptide A516, alliin, 12-HETE) and 3 clinical risk factors (medical history, HDL, FBG).

A multi-step machine learning strategy was applied to refine the 12 differential metabolites and determine the optimal feature set for TRD diagnosis (**Figure 4**). First, the Boruta algorithm was used for iterative feature selection, which filtered out 10 candidate metabolic biomarkers with robust predictive value (green features) by eliminating non-informative variables. Next, random forest model was employed to evaluate the relative importance of these 10 candidates, and eXtreme Gradient Boosting (XGBoost) was used for variable set scoring to assess the diagnostic performance of models with incrementally added markers. The model performance curve revealed that the area under the receiver operating characteristic curve (AUC) reached a stable peak when 8 metabolic markers were included, with no further significant improvement upon adding additional variables. Thus, oleamide, linoleamide (18:2n6), palmitamide (16:0), glucuronide of C10H18O2, beta-cryptoxanthin, Fibrinopeptide A516, alliin, and 12-HETE were finally locked as the core metabolic biomarkers for unimodal model construction. Additionally, correlation analysis between the 8 core metabolic markers and 3 clinical risk factors showed moderate pairwise correlations, confirming that the two feature sets could provide complementary diagnostic information and justifying their integration for dual-modal modeling.

### 3.5 Performance evaluation of unimodal diagnostic models for TRD

**Figure 5:**
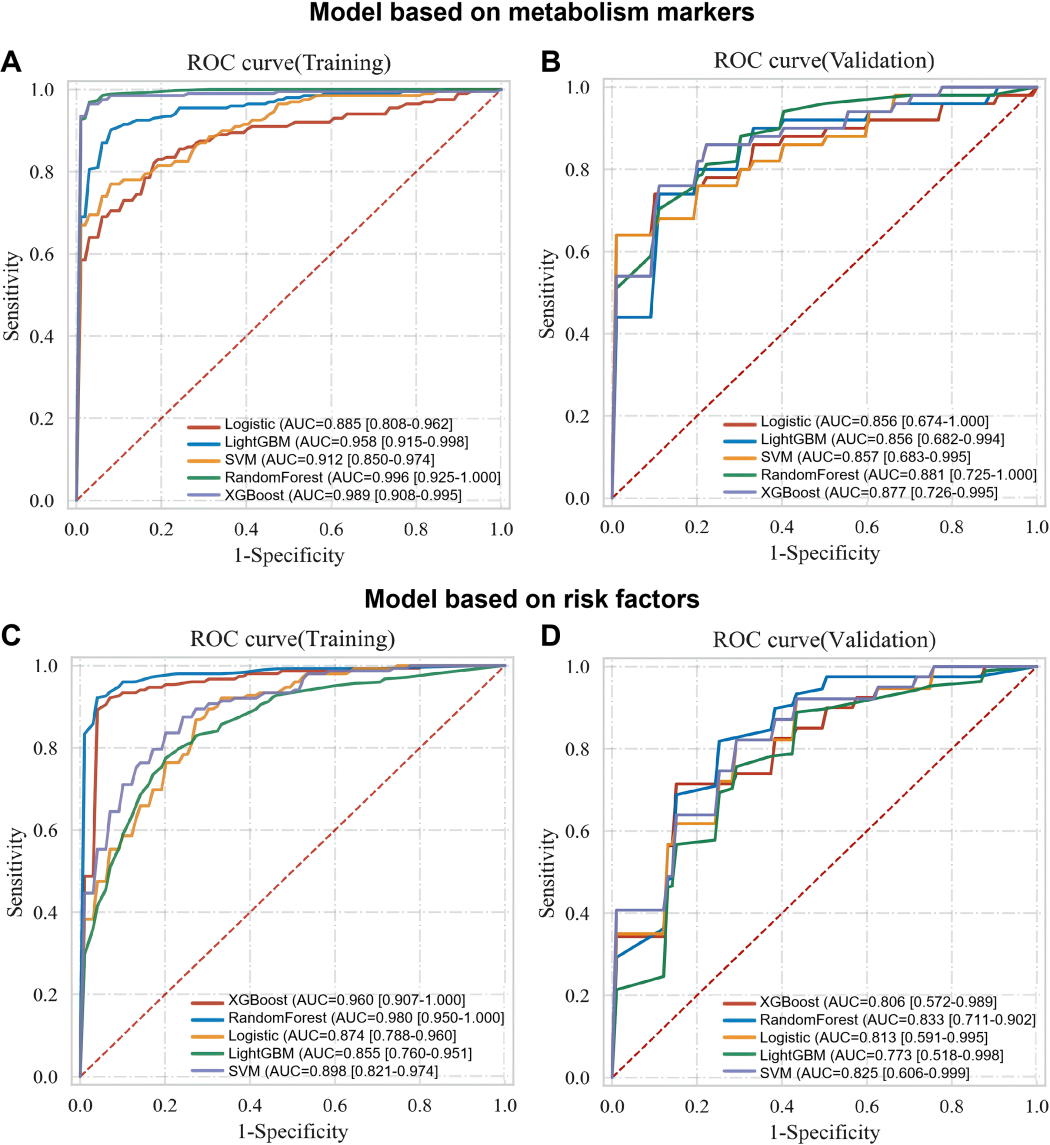
ROC curves of uni-modal TRD diagnostic models with different machine learning algorithms. ROC curves of metabolomic **(A, B)** and clinical **(C, D)** uni-modal models constructed by logistic regression, SVM, LightGBM, XGBoost and random forest in the training and validation sets.

To identify the optimal algorithm for TRD diagnosis, the 93 patients were randomly divided into a training set (70%) and a validation set (30%) using stratified random sampling (to maintain the TRD/MDD ratio). Five machine learning algorithms including logistic regression, support vector machine (SVM), LightGBM, XGBoost, and random forest were separately applied to construct two uni-modal diagnostic models based on the 8 core metabolic markers and 3 clinical risk factors, with model performance comprehensively evaluated by AUC, sensitivity, and specificity (**Figure 5**). In the training set, all algorithms achieved relatively high AUC values for both unimodal models (range: 0.856-0.996), indicating good fitting ability. However, in the independent validation set, the random forest algorithm exhibited the optimal generalization ability, with an AUC of 0.881 that was significantly higher than those of the other four algorithms (AUC range: 0.773-0.877) for both unimodal models. Based on these results, the random forest algorithm was determined as the optimal modeling method for subsequent dual-modal diagnostic model construction.

### 3.6 Performance and interpretability of the dual-modal joint diagnostic model for TRD

**Figure 6:**
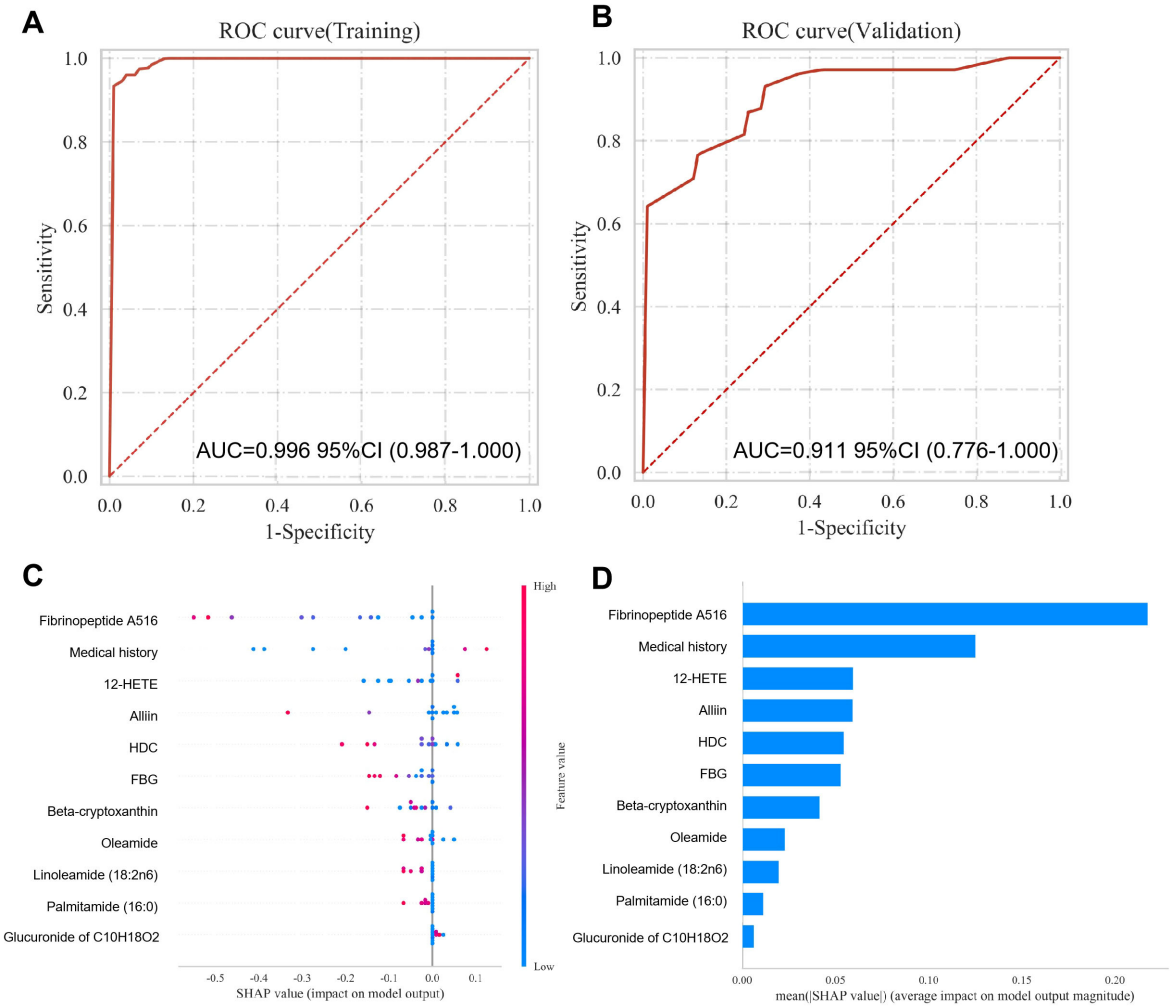
Performance and SHAP interpretability of the random forest-based dual-modal TRD diagnostic model. **(A)** Training set ROC curve (AUC = 0.996, 95%CI: 0.987-1.000); **(B)** Validation set ROC curve (AUC = 0.911, 95%CI: 0.776–1.000); **(C)** SHAP summary plot showing the impact of each feature on model output; **(D)** Bar plot of mean absolute SHAP values ranking the core predictive features for the model.

A dual-modal joint diagnostic model was constructed by integrating the 8 core metabolic markers and 3 clinical risk factors based on the random forest algorithm, with its diagnostic performance and feature interpretability systematically analyzed (**Figure 6**; **Table 2**). The model showed excellent diagnostic efficacy in both the training and validation sets: the AUC reached 0.996 (95%CI: 0.987-1.000) in the training set, and maintained a high performance of 0.911 (95%CI: 0.776-1.000) in the independent validation set, which was significantly higher than the AUC of either single-modal model. SHAP (SHapley Additive Explanations) analysis was further performed to interpret the black-box characteristics of the random forest model: the SHAP summary plot and mean absolute SHAP value bar chart clearly identified Fibrinopeptide A516, 12-HETE, and the three clinical risk factors (medical history, HDL, FBG) as the core driving features of the model. The model showed excellent calibration (Brier score = 0.0168, **Supplementary Figure 1A**). Decision curve analysis further demonstrated positive net benefit across clinically relevant threshold probabilities (0.05-0.85) compared with treat-all or treat-none strategies (**Supplementary Figure 1B**). These results quantitatively characterized the direction and magnitude of each feature’s impact on the model’s TRD prediction, clarifying the key contributors to the high diagnostic performance of the dual-modal model.

**Table 2:** The performance of the random forest-based dual-modal TRD diagnostic model.

Dataset	AUC (95%CI)	Cutoff (95%CI)	Accuracy (95%CI)	Sensitivity (95%CI)	Specificity (95%CI)	F1 (95%CI)	Kappa (95%CI)
Training	0.996 (0.987-1.000)	0.6 (0.538-0.662)	0.959 (0.947-0.971)	0.925 (0.898-0.952)	0.995 (0.984-1.005)	0.958 (0.945-0.972)	0.918 (0.894-0.943)
Validation	0.911 (0.776-1.000)	0.592 (0.550-0.633)	0.855 (0.842-0.867)	0.755 (0.745-0.765)	0.958 (0.928-0.989)	0.841 (0.830-0.853)	0.71 (0.685-0.736)

To ensure that the high diagnostic performance reported in the primary 7:3 validation was not driven by information leakage or overfitting, we performed nested cross-validation as a secondary validation strategy. Bootstrap stability analysis further showed that all 8 core metabolic markers and 3 clinical risk factors were selected in >70% of resamplings (**Supplementary Table 1**). The nested CV yielded a mean AUC of 0.949 (95% CI: 0.866–0.997), confirming that the model maintains robust discrimination when all feature selection is strictly confined to training folds (**Supplementary Table 3**).

### 3.7 KEGG pathway enrichment analysis of differential metabolites

**Figure 7:**
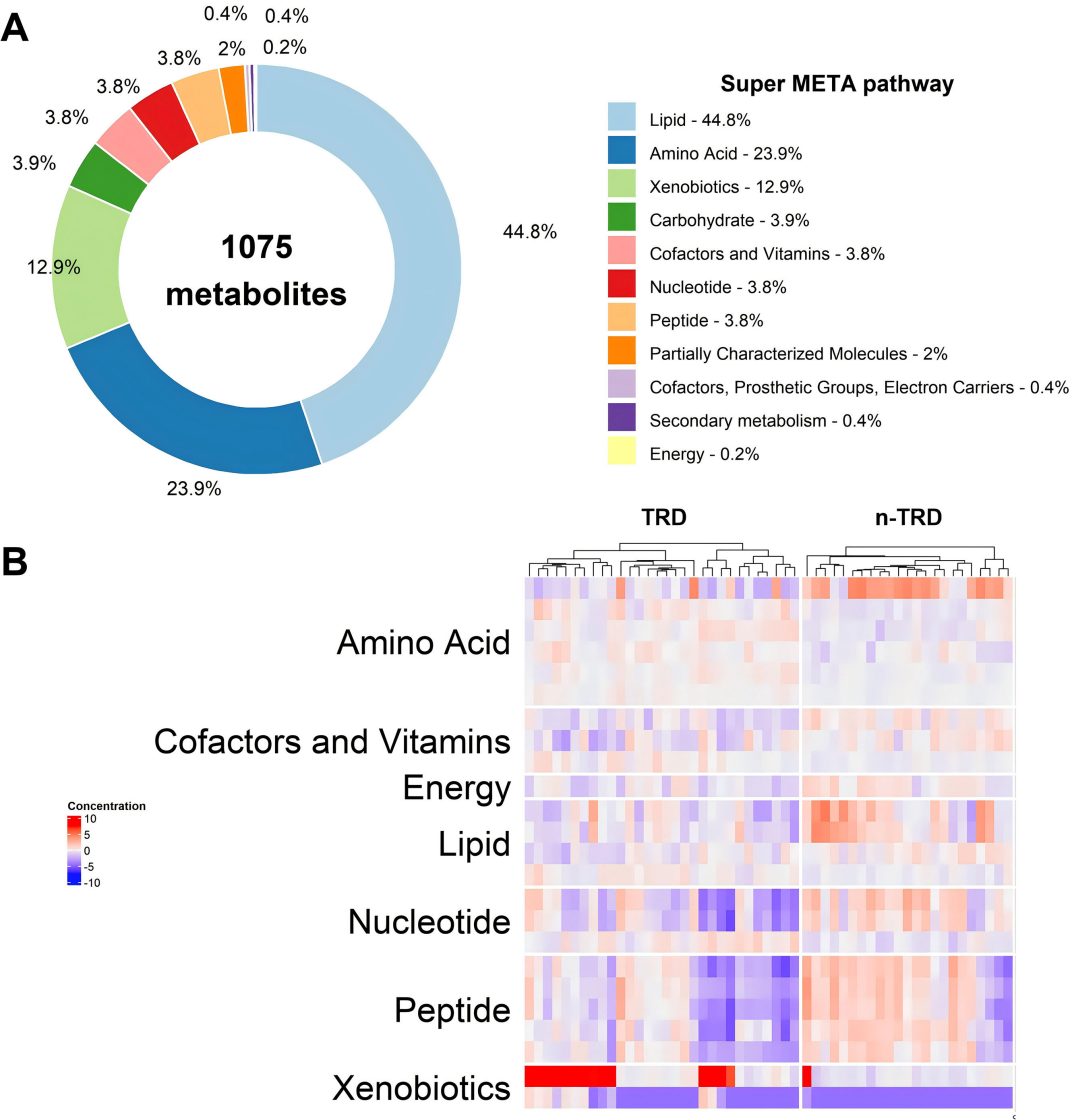
KEGG pathway enrichment analysis of differential metabolites between TRD and n-TRD groups. **(A)** Proportional distribution of differential metabolites in enriched metabolic pathways [dominated by lipid metabolism (44.8%) and amino acid metabolism (23.9%)]; **(B)** Log2(fold change) analysis of key metabolites in major enriched pathways for TRD and MDD groups.

To explore the potential pathophysiological mechanisms of TRD associated with metabolic perturbations, KEGG pathway enrichment analysis was performed on the 12 significantly differential metabolites (with valid KEGG IDs) using MetPA and Pathview toolkits (**Figure 7**). The enrichment results showed that the differential metabolites were mainly enriched in three major metabolic pathways: lipid metabolism (44.8%), amino acid metabolism (23.9%), and xenobiotic metabolism (12.9%), with minor enrichment in carbohydrate metabolism, nucleotide metabolism, and peptide metabolism. Further analysis of the expression levels of key metabolites in these enriched pathways confirmed distinct metabolic dysregulation between the TRD and MDD groups. Notably, the enrichment of differential metabolites in lipid and amino acid metabolic pathways, as well as inflammation-related metabolic processes, suggested that lipid metabolism disorder, amino acid metabolism imbalance, and chronic inflammatory response may be closely involved in the pathophysiological process of treatment resistance in depression, providing novel metabolomic insights into the molecular mechanisms of TRD.

## 4 Discussion

MDD represents a substantial global health burden, yet approximately one-third of patients develop TRD following standard antidepressant therapy, facing prolonged suffering and increased healthcare costs. The current clinical diagnosis of TRD relies exclusively on retrospective assessment of treatment response and subjective symptom evaluation, lacking objective biomarkers to guide early identification and intervention. In this study, we integrated untargeted serum metabolomics with routine clinical indicators and employed a multi-step machine learning strategy to construct a dual-modal diagnostic model for TRD. Our results demonstrated that the random forest-based model combining eight core metabolic biomarkers and three clinical risk factors achieved an AUC of 0.911 (95%CI: 0.776–1.000) in the independent validation set, significantly outperforming single-modal approaches. These findings provide a novel, quantifiable tool for objective TRD identification and offer insights into the metabolic pathophysiology underlying treatment resistance.

The eight core metabolic biomarkers identified through our multi-step feature selection strategy reveal distinct biological processes implicated in TRD pathogenesis. Notably, three endogenous fatty acid amides-oleamide, linoleamide (18:2n6), and palmitamide (16:0)-were significantly downregulated in TRD patients serum. These lipid mediators function as critical signaling molecules within the endocannabinoid system (ECS), modulating neuroinflammation, synaptic plasticity, sleep-wake cycles, and stress responses (9–11). Reduced circulating levels of these amides suggest impaired ECS signaling in treatment-resistant depression, potentially reflecting chronic depletion of neuroprotective lipid mediators due to sustained stress, inflammation, or prolonged disease duration (12). Preclinical studies have demonstrated that oleamide possesses anxiolytic, sleep-promoting, and anti-inflammatory properties; its deficiency may compromise the endogenous mechanisms that normally limit neuroinflammatory responses and maintain synaptic homeostasis (13–15). Our findings extend these observations to the clinical context of TRD, suggesting that ECS hypofunction—rather than hyperactivation—characterizes treatment-resistant cases, aligning with the “clinical endocannabinoid deficiency syndrome” hypothesis proposed in chronic stress-related disorders (16).

Conversely, the elevation of 12-hydroxyeicosatetraenoic acid (12-HETE) and fibrinopeptide A in TRD patients supports the involvement of pro-inflammatory and pro-thrombotic processes in treatment resistance (17, 18). 12-HETE, a product of arachidonic acid metabolism via 12-lipoxygenase, exhibits potent pro-inflammatory properties and has been implicated in blood-brain barrier dysfunction (19, 20). The reciprocal pattern of reduced anti-inflammatory amides and elevated pro-inflammatory eicosanoids suggests a disrupted lipid mediator balance in TRD, favoring neuroinflammatory and pro-oxidative states. This imbalance characterized by deficiency of endogenous anti-inflammatory counter-regulatory mechanisms alongside activation of innate immune responses may constitute a metabolic signature of treatment refractoriness. Fibrinopeptide A, released during thrombin-mediated fibrinogen cleavage, indicates activation of the coagulation cascade, which interacts with neuroinflammatory pathways and may contribute to cerebrovascular dysfunction in refractory depression.

The reduction in beta-cryptoxanthin, a dietary carotenoid with antioxidant and anti-inflammatory properties, further supports the presence of compromised antioxidant defenses in TRD (21). This finding aligns with accumulating evidence linking oxidative stress and reduced antioxidant capacity to depression severity and treatment resistance (22, 23). The concomitant decrease in alliin, a compound associated with gut microbiota metabolism and sulfur-containing amino acid pathways, hints at potential alterations in gut-brain axis communication and microbial-host metabolic crosstalk in treatment resistance (24, 25). The glucuronide conjugate of C10H18O2, while structurally requiring further validation, may represent altered xenobiotic metabolism or phase II detoxification processes. Collectively, these metabolic alterations depict TRD as a state characterized by deficiency of neuroprotective and anti-inflammatory mediators alongside activation of pro-inflammatory and pro-oxidative pathways. This metabolic phenotype may impede therapeutic response.

The three identified clinical risk factors (medical history, HDL, FBG) are readily accessible and have important clinical and pathological significance for TRD. Low HDL is associated with chronic low-grade inflammation and impaired reverse cholesterol transport, and as a carrier for ECS lipid mediators (26), its reduction further exacerbates ECS hypofunction in TRD. Reduced FBG in the TRD group may reflect metabolic dysregulation or altered glucose homeostasis associated with chronic stress and depressive pathology, which has been linked to poor antidepressant response. Longer medical history reflects the accumulation of biological alterations such as progressive ECS dysfunction and inflammatory burden in chronic TRD cases. These factors enable initial clinical risk stratification for TRD, forming a simple and feasible preliminary screening tool for clinical practice.

It is critical to emphasize that the cross-sectional design and machine learning framework employed here identify statistical associations, not causal relationships. The metabolic alterations observed may represent consequences of treatment resistance, compensatory responses, or epiphenomena of chronic illness. Longitudinal and interventional studies are required to disentangle these possibilities and to validate whether the identified pathways are causally involved in treatment refractoriness.

The integration of metabolic biomarkers and clinical risk factors is the key to the superior diagnostic performance of the dual-modal model, as the two data types exhibit complementary advantages. Metabolomic profiling captures the intrinsic pathophysiological state of TRD patients, reflecting the downstream effects of genetic, environmental and microbial factors, while clinical risk factors provide rapid and accessible information on disease chronicity and metabolic comorbidities. SHAP analysis confirmed that Fibrinopeptide A516, 12-HETE and the three clinical factors jointly drive model predictions, with no single feature dominating classification. Methodologically, the multi-step machine learning pipeline effectively minimized overfitting, and the random forest algorithm ensured good generalization ability. This dual-modal strategy establishes a tiered diagnostic approach for TRD, and the identified ECS hypofunction also provides a promising therapeutic target—preclinical studies have shown that ECS signaling enhancement exerts antidepressant-like effects (27), suggesting potential for TRD targeted therapy.

Recent efforts in precision psychiatry have increasingly relied on multi-modal data integration to improve depression prediction beyond single-modality approaches. For instance, Zha et al. constructed a machine learning-based predictive model for depression in patients with advanced cardiovascular-kidney-metabolic syndrome by integrating sleep disorders, waist circumference, and gamma-glutamyl transferase with demographic features, achieving an AUC of 0.768 in the test set and consistent performance across three external validation cohorts (AUC 0.765–0.794) (28). While these studies demonstrate the feasibility of combining clinical and biological variables for risk stratification, they predominantly employ structured clinical data or targeted laboratory panels rather than untargeted metabolomics that captures the global metabolic network state. Our study addresses this methodological gap by integrating serum untargeted metabolomics with routine clinical indicators and employing SHAP-based interpretability to dissect the relative contributions of metabolic versus clinical drivers, thereby moving beyond “black-box” prediction toward mechanistically informed biomarker panels. This distinction is particularly relevant given the growing emphasis on metabolomics as the functional endpoint of gene-environment interactions in psychiatric disorders. Cavaleri et al. recently underscored that metabolomic biomarkers offer actionable targets for individualized treatment in precision psychiatry (29). our identified endocannabinoid deficiency, inflammatory lipid mediator imbalance, and antioxidant depletion align closely with this framework. Furthermore, the clinical utility of objective TRD identification extends beyond diagnosis to therapeutic stratification. Sun et al. reviewed the emerging evidence for transcutaneous auricular vagus nerve stimulation as a non-invasive intervention for MDD (30), whereas Wang et al. demonstrated the efficacy of school-based narrative writing interventions for adolescent anxiety and depression in a large cluster RCT (31). These advances in therapeutic modalities highlight the unmet need for biomarker-guided patient selection-an objective that our dual-modal model is positioned to address in future prospective trials.

We acknowledge that the primary validation relied on a single-center internal dataset with a modest sample size, and that multi-step feature selection in high-dimensional metabolomic data carries inherent risks of overfitting. To address this transparently, we performed nested cross-validation, which yielded a mean AUC of 0.949. This convergence between the primary and secondary validation estimates suggests that the original performance metrics were not substantially inflated by information leakage. Furthermore, bootstrap resampling confirmed high feature selection stability for the core biomarkers, supporting the reproducibility of the identified metabolic signature. LASSO-regularized regression independently retained the three clinical risk factors, confirming their discriminative value beyond univariate associations. Decision curve analysis demonstrated positive net benefit across clinically relevant threshold probabilities, providing preliminary evidence of potential clinical utility pending external validation.

This study has several limitations that need to be acknowledged. First, this study represents a single-center, proof-of-concept investigation with a modest sample size (n=93). While the nested cross-validation framework provides the most rigorous internal validation possible with available data, we explicitly acknowledge that external validation in independent multi-center cohorts is mandatory before any clinical deployment. Second, the TRD definition based on HAMD-17 criteria does not capture functional recovery and medication adherence, potentially introducing diagnostic bias. Third, the cross-sectional design precludes determining the causal relationship between metabolic alterations and TRD, and some metabolites require further structural validation. Future research should prioritize longitudinal investigations to develop TRD treatment response prediction models, mechanistic studies using cellular/animal models to verify ECS hypofunction and oleamide deficiency, and integration of metabolomic data with pharmacogenomic and microbiome profiling. Most importantly, intervention studies targeting the identified metabolic pathways are needed to develop novel mechanism-based treatment strategies for TRD.

## 5 Conclusion

In conclusion, this study demonstrates that integrating serum metabolomic profiling with routine clinical parameters through machine learning enables robust, objective identification of treatment-resistant depression. The identified metabolic signatures implicate endocannabinoid deficiency, inflammatory lipid mediator imbalance, and antioxidant depletion in TRD pathophysiology, providing a foundation for both improved diagnostic precision and mechanism-based therapeutic development. As psychiatry moves toward precision medicine approaches, multi-modal biomarker panels such as the one developed here offer a pathway to reduce the empirical nature of depression treatment and improve outcomes for this challenging patient population.

## Data Availability

The raw data supporting the conclusions of this article will be made available by the authors, without undue reservation.
