# Supplementary material for: Precision diagnosis model for treatment-resistant depression integrating serum metabolomics and clinical risk factors
Source: Front Psychiatry. 2026 May 21;17:1826019. doi: 10.3389/fpsyt.2026.1826019 (PMC13233471; doi:10.3389/fpsyt.2026.1826019)
Supplement: Supplementary file 1 [file DataSheet1.docx]

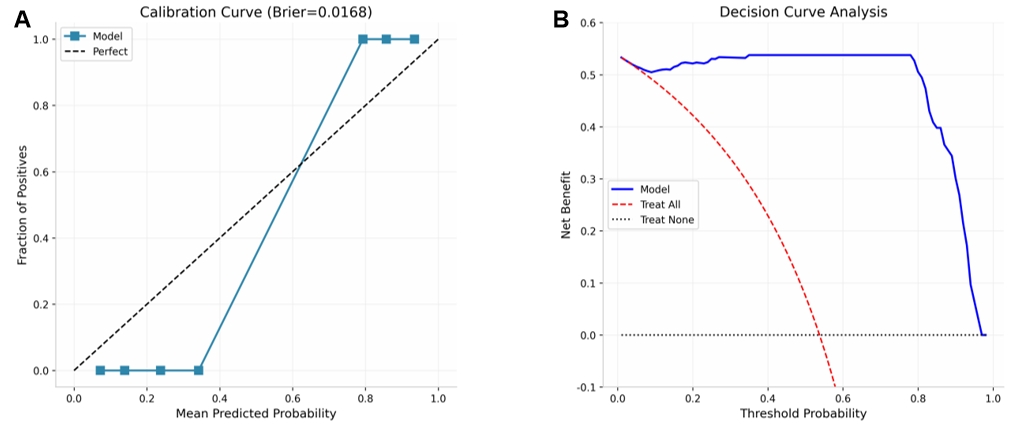


Figure S1. Secondary validation analyses of the dual-modal random forest model. (A) Calibration curve showing the agreement between predicted probabilities and observed outcomes (Brier score = 0.0168). (B) Decision curve analysis (DCA) comparing the net benefit of the dual-modal model against treat-all and treat-none strategies across clinically relevant threshold probabilities (0.05-0.85).

Table S1. Bootstrap stability analysis of candidate features across 100 resampling iterations.

| Feature | Stability (n=100) | >70% |
| --- | --- | --- |
| Oleamide | 0.79 | Yes |
| Alliin | 0.76 | Yes |
| Linoleoylglycerol | 0.38 | No |
| Glycylvaline | 0.15 | No |
| 12-HETE | 0.97 | Yes |
| Linoleamide (18:2n6) | 0.71 | Yes |
| Oleoylglycerol | 0.04 | No |
| Hydroxyoctanoylcarnitine | 0.31 | No |
| palmitamide | 0.71 | Yes |
| Beta-cryptoxanthin | 0.94 | Yes |
| Fibrinopeptide A516 | 1.00 | Yes |
| Glucuronide of C10H18O2 | 0.75 | Yes |
| Sex | 0 | No |
| Age | 0.27 | No |
| Medical history | 0.75 | Yes |
| HDL | 0.91 | Yes |
| FBG | 0.76 | Yes |

Table S2. LASSO-regularized multivariable logistic regression coefficients and retention status.

| Variable | Coefficient | Retained |
| --- | --- | --- |
| Glycylvaline | 0 | No |
| Alliin | -0.4192 | Yes |
| Linoleoylglycerol | 0 | No |
| Oleamide | -0.2558 | Yes |
| 12-HETE | -0.0272 | Yes |
| Oleoylglycerol | 0 | No |
| Linoleamide (18:2n6) | -0.3434 | Yes |
| Hydroxyoctanoylcarnitine | 0.2858 | Yes |
| Palmitamide | -0.3187 | Yes |
| Beta-cryptoxanthin | -1.4089 | Yes |
| Fibrinopeptide A516 | -0.9529 | Yes |
| Glucuronide of C10H18O2 | 3.3492 | Yes |
| Sex | 0 | No |
| Age | 0 | No |
| Medical history | 1.0136 | Yes |
| HDL | -1.1413 | Yes |
| FBG | -1.9467 | Yes |

Table S3. Nested 5×3-fold cross-validation results of the dual-modal random forest model.

| Fold | Train N | Test N | Best mtry | Inner AUC | Outer AUC |
| --- | --- | --- | --- | --- | --- |
| 1 | 74 | 19 | 2 | 0.9495 | 0.9556 |
| 2 | 74 | 19 | 2 | 0.9533 | 0.8556 |
| 3 | 74 | 19 | 2 | 0.9195 | 0.9611 |
| 4 | 75 | 18 | 2 | 0.9369 | 0.975 |
| 5 | 75 | 18 | 2 | 0.893 | 1 |
|  |  |  |  |  |  |
| Mean |  |  |  |  | 0.9494 |
| SD |  |  |  |  | 0.0494 |
| 95% CI Lower |  |  |  |  | 0.8656 |
| 95% CI Upper |  |  |  |  | 0.9975 |
